# Transactions of the Associations of American Physicians

**Published:** 1887-08

**Authors:** 


TRANSACTIONS OF THE ASSOCIATION OF AMERICAN PHYSICIANS. First Session, Washington, D. C., June 17 and 18, 1886. Francis Delafield, M. D., President; James Lynn, M. D., Secretary; James T. Whitaker, M. D., Recorder. Philadelphia: Wm. J. Dorman, Printer. 1886.

It will be noted that the number of members is limited to one hundred, and the number of honorary members does not exceed twenty-five; while only seventy-five on the former list and seven on the latter are recorded for the first year's operations. Doubtless ere this all the available places have been filled, unless it turns out that "Physicians of sufficient eminence to merit the distinction" cannot be found among the eighty thousand doctors scattered throughout the United States. The self-elected original constituents of this association are for the most part well-known medical men, but it is quite certain that another lot of seventy-five could readily be found who might claim equal prominence with them, and be recognized as entitled, by their productions, to equal consideration at home and abroad as physicians. It is most surprising that the association assumes no responsibility for the statements and opinions published in this volume of transactions, containing the papers read before the body, since it might naturally have been inferred that such contributions would prove unexceptionable. The very thought of undertaking to criticize the papers presented at the meeting makes one's head dizzy, and though subjects of vital importance are treated elaborately and discussed thoroughly, the apprehension of displaying rashness or being liable to the charge of impertinence must preclude any commentary upon their contents.

Among other highly important topics there are contributions on chronic catarrhal gastritis, tendon and muscle-jerk, method of managing typhoid fever, correlative of gout, rheumatism, diabetes and chronic Bright's disease, spasm of the glottis, in rickets, cases of diaphragmatic pleurisy, elements of blood in malarial fever, perforating inflammation of the vermiform appendix, periuterine inflammation, glomerulo-nephritis, bicuspid condition of the aortic valves, and the bacillus of typhoid fever. They may all be read with profit as emanating from representative men in special departments of investigation.

